# HIV Infection Is Associated with a Lower Incidence of Constriction in Presumed Tuberculous Pericarditis: A Prospective Observational Study

**DOI:** 10.1371/journal.pone.0002253

**Published:** 2008-06-04

**Authors:** Mpiko Ntsekhe, Charles S. Wiysonge, Freedom Gumedze, Gary Maartens, Patrick J. Commerford, Jimmy A. Volmink, Bongani M. Mayosi

**Affiliations:** 1 Department of Medicine, University of Cape Town, Cape Town, South Africa; 2 South African Cochrane Centre, Medical Research Council, Cape Town, South Africa; 3 Department of Statistical Sciences, University of Cape Town, Cape Town, South Africa; 4 Faculty of Health Sciences, University of Stellenbosch, Tygerberg, South Africa; University of Stellenbosch, South Africa

## Abstract

**Background:**

Pericardial constriction is a serious complication of tuberculous pericardial effusion that occurs in up to a quarter of patients despite anti-tuberculosis chemotherapy. The impact of human immunodeficiency virus (HIV) infection on the incidence of constrictive pericarditis following tuberculous pericardial effusion is unknown.

**Methods and Results:**

We conducted a prospective observational study to determine the association between HIV infection and the incidence of constrictive pericarditis among 185 patients (median age 33 years) with suspected tuberculous pericardial effusion. These patients were recruited consecutively between March and October 2004 on commencement of anti-tuberculosis treatment, from 15 hospitals in Cameroon, Nigeria and South Africa. Surviving patients (N = 119) were assessed for clinical evidence of constrictive pericarditis at 3 and 6 months of follow-up. Clinical features of HIV infection were present in 42 (35.2%) of the 119 patients at enrolment into the study. 66 of the 119 (56.9%) patients consented to HIV testing at enrolment. During the 6 months of follow-up, a clinical diagnosis of constrictive pericarditis was made in 13 of the 119 patients (10.9 %, 95% confidence interval [CI] 5.9–18%). Patients with clinical features of HIV infection appear less likely to develop constriction than those without (4.8% versus 14.3%; P = 0.08). None of the 33 HIV seropositive patients developed constriction, but 8 (24.2%, 95%CI 11.1–42.3%) of the 33 HIV seronegative patients did (P = 0.005). In a multivariate logistic regression model adjusting simultaneously for several baseline characteristics, only clinical signs of HIV infection were significantly associated with a lower risk of constriction (odd ratio 0.14, 95% CI 0.02–0.87, P = 0.035).

**Conclusions:**

These data suggest that HIV infection is associated with a lower incidence of pericardial constriction in patients with presumed tuberculous pericarditis.

## Introduction

Constrictive pericarditis is a serious complication of tuberculous pericarditis that is associated with high morbidity and mortality of 3–15% following pericardiectomy [1]. Prior to the introduction of effective anti-tuberculosis chemotherapy, up to 50% of patients with effusive tuberculous pericarditis progressed to the constrictive stage of the disease [2]. The introduction of rifampicin-based anti-tuberculosis treatment in the 1970's resulted in the reduction of the incidence of constriction to about 25% in patients with effusive tuberculous pericarditis [3], [4]

In the last two decades, the human immunodeficiency virus (HIV) pandemic has had a significant effect on the epidemiology of pulmonary and extra-pulmonary tuberculosis, but the impact of HIV infection on the development of pericardial fibrosis in tuberculous pericarditis is not known [5]. This question is relevant because IL-13-secreting CD4^+^ T_H_2 cells, which are reduced by HIV, regulate fibrogenesis directly, through stimulating collagen synthesis by fibroblasts and indirectly by promoting TGF-β1 production by macrophages [6]. Furthermore, fewer granulomata have been observed in HIV infected tuberculous pericarditis patients with severely depleted CD4 lymphocytes, suggesting that there may be less propensity to develop pericardial fibrosis in HIV-infected individuals with tuberculous pericardial effusion [7].

We hypothesised that people with HIV-associated tuberculous pericardial effusion may have a lower incidence of pericardial constriction than those with pericardial tuberculosis and no evidence of HIV infection during 6 months of anti-tuberculosis chemotherapy. We tested this hypothesis by assessing the incidence of clinical signs of constrictive pericarditis in survivors of tuberculous pericardial effusion who were enrolled in the *I*nvestigation of the *M*anagement of *P*ericarditis in *A*frica (IMPI Africa) registry [8].

## Methods

The IMPI Africa registry was a multi-centre, prospective, observational study of patients with presumed tuberculous pericarditis who were admitted consecutively to 15 referral hospitals in Cameroon, Nigeria, and South Africa. The project was approved by the local research ethics committees (listed at the end of the paper) and all participants gave written informed consent. The study was designed to assess the clinical presentation, initial management, and outcome of patients with suspected tuberculous pericarditis in the HIV era. The design of the study and baseline characteristics of the participants enrolled in the registry are described in detail elsewhere [8]. Briefly, the participants were recruited between March 1, 2004 and October 31, 2004. The diagnosis of tuberculous pericarditis was determined by clinical assessment, and diagnostic evaluation generally included ultrasound, electrocardiogram, and chest X-ray. In keeping with the observational nature of the study and the prespecified aim of documenting physician practice across the sub-continent, no attempt was made to standardize the diagnostic approach or diagnostic procedures and microbiological confirmation was conducted at the discretion of the attending physician. On enrolment into the study, clinical features of human immunodeficiency virus (HIV) infection were determined by standard criteria [9] and HIV serological testing was offered, where possible, following voluntary counselling. The CD4 T cell count was not recorded in this registry.

Patients with effusive pericardial disease on enrolment were assessed clinically at three and six months of follow-up for clinical evidence of constrictive pericarditis, which was defined as persistent signs of elevated systemic venous pressure in the absence of a pericardial effusion [10] The finding of a clinical diagnosis of constrictive pericarditis at either the 3 month or 6 month follow up visit constituted the outcome of interest. Only those patients with no clinical evidence of constriction at 3 months were reassessed for evidence of constriction at 6 months.

Data were analysed using Epi Info 3.3.2 (CDC, Atlanta, GA). We used the Chi-squared test or Fisher's exact test (as appropriate) to assess probabilities of significant differences between proportions and logistic regression to test which factors among presence of clinical signs of HIV infection, age, sex, or cardiac tamponade were associated with constrictive pericarditis at 6 months of follow-up. Serological HIV status could not fit into the logistic regression model since none of the HIV seronegative patients developed constriction. All significance tests were two-tailed and statistical significance was defined at the alpha level of 0.05.

## Results

**Figure 1:**
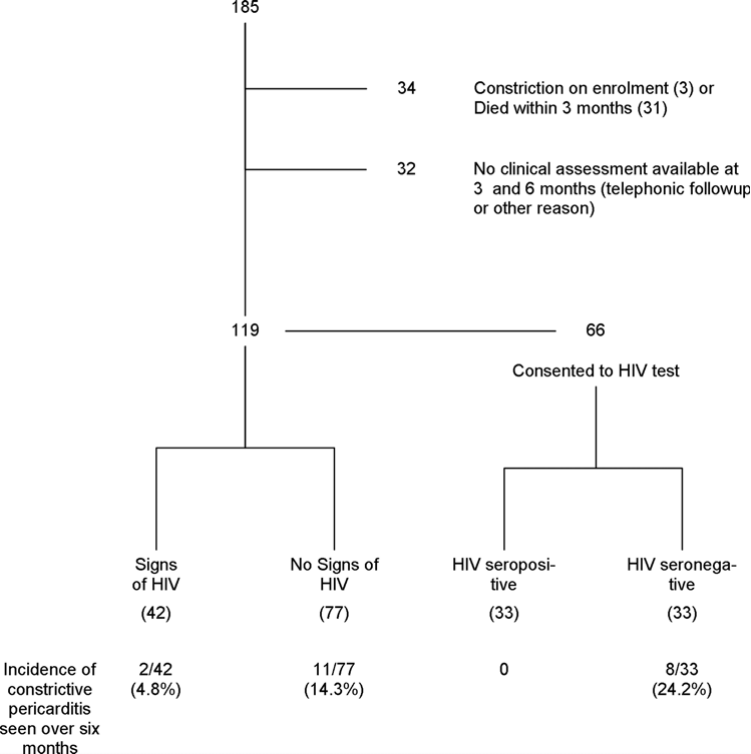
Study flow chart of patients with presumed tuberculous pericarditis.

185 patients with presumed tuberculous pericarditis (median age 33, range 14–87 years) were studied (Fig. 1). On enrolment, there were three patients out of 185 (1.6%) who were diagnosed with constrictive pericarditis, none of whom had clinical features of HIV infection; one of the three consented to HIV testing and tested negative. These three patients were excluded from the current analysis. An additional sixty-three patients were excluded from the analysis for the following reasons: (i) death before clinical assessment at three or six months (N = 31); (ii) follow-up was either telephonic or the clinical assessment was not possible for other reasons (N = 32) (Fig. 1). There were no significant differences in the baseline clinical characteristics between the patients who were excluded and those who were included in the prospective analysis of incidence and determinants of pericardial constriction (Table 1).

**Table 1:** Comparison of baseline characteristics of patients included and excluded from analysis.

Characteristic	Excluded (n = 66)	Included (n = 119)	P-value
Age median (range), years	33 (14–69)	33 (15–87)	0.178
Gender			
* Female*	29 (43.9)	53 (44.5)	
* Male*	37 (56.1)	66 (55.5)	0.937
Clinical features of HIV infection			
* Yes*	32 (48.5)	42 (35.2)	
* No*	34 (51.5)	77 (64.7)	0.079
Serological HIV			
* Yes*	20 (66.7)	33 (50.0)	
* No*	10 (33.3)	33 (50.0)	0.128
NYHA++ Functional Class			
* I*	10 (15.2)	29 (24.3)	
* II*	25 (37.9)	44 (37.0)	
* III*	16 (24.2)	31 (26.1)	
* IV*	15 (22.7)	15 (12.6)	0.216
Pericardiocentesis			
* Yes*	1 (14.3)	25 (28.4)	
* No*	6 (85.7)	63 (71.6)	0.420
Adjunctive steroid use			
* Yes*	37 (56.1)	72 (60.5)	
* No*	29 (43.9)	47 (39.5)	0.556
Haemodynamic instability**			
* Yes*	23 (34.9)	31 (26.1)	
* No*	43 (65.1)	88 (74.0)	0.207

Values are median (range) and absolute counts (percentages);

++ NYHA, New York Heart Association (I, No limitation of physical activity; II, Slight limitation of physical activity; III, Marked limitation of physical activity; and IV, Unable to carry out any physical activity without discomfort);

** Pulse rate more than 100 bpm, Systolic blood pressure less than 100 mmHg and or tamponade requiring centesis.

**Figure 2:**
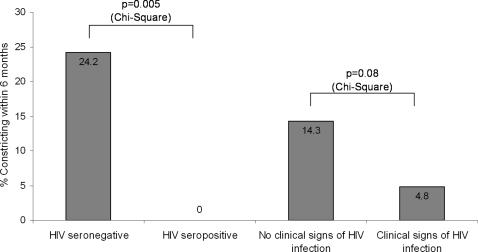
A summary of the incidence of clinical constriction categorised by clinical features of HIV infection and by HIV sero-status.

Overall, a clinical diagnosis of constrictive pericarditis was made in 13/119 participants who were followed up and assessed clinically at three and six months (10.9%; 95% confidence interval [CI] 5.9–18%) (Fig. 1). Patients with clinical features of HIV infection were less likely than those without to develop constriction (4.8% [2/42] versus 14.3% [11/77]; P = 0.08) (Fig. 1, Fig. 2). Sixty six of the 119 patients consented to HIV testing on enrolment into the study, 33 (50%) of whom were HIV sero-positive. None of the HIV positive patients developed constriction compared to 8 (24.2%) HIV negative patients (P = 0.005) (Fig. 1, Fig. 2).

We evaluated whether the following baseline characteristics were associated with the risk of constrictive pericarditis: age, sex, HIV infection, pericardiocentesis, adjunctive steroid use, and haemodynamic instability at enrolment (Table 2). In the univariate analysis, clinical evidence of HIV infection was the factor which approached statistical significance (P = 0.08). We constructed a multivariate logistic regression model to adjust simultaneously for all the above factors, and found only clinical evidence of HIV infection was associated with a reduced risk of constriction (odd ratio 0.14, 95% CI 0.02–0.87, P = 0.035) (Table 2).

**Table 2:** Logistic regression analyses to determine predictors of constriction

Baseline characteristic	Univariate analysis			Multivariate analysis		
	OR	95% CI	P	OR	95% CI	P
Age	1.00	0.96–1.05	0.965	1.02	0.97–1.06	0.442
Men	1.66	0.53–5.17	0.386	1.59	0.44–5.77	0.479
Clinical signs of HIV infection	0.25	0.05–1.18	0.081	0.14	0.02–0.87	0.035
Pericardiocentesis	2.00	0.60–7.02	0.279	2.36	0.57–9.80	0.236
Adjunctive steroid use	0.74	0.23–2.34	0.604	0.31	0.07–1.32	0.113
Haemodynamic instability*	0.70	0.18–2.65	0.596	0.52	0.11–2.39	0.403

OR, Odds ratio; CI, Confidence interval; P, the probability that the effect of the characteristic on constriction in this study occurred by chance alone, given that there is truly no relationship between the characteristic and re-admission to hospital;

* Pulse rate more than 100 beats per minute, Systolic blood pressure less than 100 mmHg and or tamponade requiring pericardiocentesis; HIV, human immunodeficiency virus;

** None of the HIV sero-positive group developed clinical features of constriction. Therefore, this factor was not entered in the logistic regression model.

## Discussion

In patients with pericardial effusion presumed to be due to tuberculosis, the presence of clinical features of HIV infection and positive HIV serology appear to be associated with a lower incidence of clinical signs of constrictive pericarditis following 3–6 months of anti-tuberculosis treatment. This study is, to the best of our knowledge, the first demonstration of this apparent protective effect of HIV infection and immmunosupression against the development of pericardial fibrosis. The observation is, however, consistent with the finding that HIV-associated pulmonary tuberculosis is associated less with lung fibrosis and cavitation, and more with a disseminated pattern of disease in patients with advanced immunosuppression [11].

Constrictive pericarditis occurs within six months in up to 25% of patients with tuberculous pericardial effusion despite treatment with anti-tuberculosis medication and corticosteroids [3], [4]. Therefore, the overall frequency of pericardial constriction of 10.9% in this registry is in the lower range of the rates that have been recorded in other prospective series. However, the frequency of constriction of 24.2% in patients known to be HIV sero-negative in our study is similar to the historic rate recorded in the pre-HIV era [3], [4]. Pericardial constriction is known to occur up to two years following an episode of effusive pericardial tuberculosis. Therefore, the short follow-up of six months may have under-estimated the incidence of constriction in our cohort, and these findings apply to constriction that develops within three to six months of follow-up.

Our study has several limitations. First, the six month mortality rate for those with clinical evidence of HIV infection was 40%, compared with 17% in those who were clinically HIV uninfected (17%), and the exact cause of death was not determined [12]. Bias will have been introduced if death intervened in the former group before patients could present with clinically detectable constrictive pericarditis. Alternatively some may have died from intractable heart failure from early constriction.

Second, the simple clinical observational design of the registry meant that the diagnosis of constriction was based on non-invasive clinical and echocardiographic criteria, and the tuberculous aetiology of the pericardial disease was confirmed only 7% of the cohort [8]. This practice, however, reflects routine clinical care in resource-poor settings of many African countries. The registry was an observational study that was designed to capture the contemporary practice and factors affecting outcome in patients who are treated for tuberculous pericarditis [8], [13].

Finally, not all patients were tested for HIV. HIV status could be confirmed in slightly more than half of the cases with no cases of constriction being observed among the few HIV seropositive patients. This precluded the inclusion of proven HIV status in the multivariate model. There was, however, internal consistency of the association of clinical signs of HIV infection and confirmed HIV infection, on the one hand, and the incidence of constriction on the other hand. The sensitivity and specificity of clinical HIV for confirmed HIV was 76% and 88%, respectively, in this study[8].

It is worthy to note that the trend towards a lower frequency of pericardial constriction in HIV positive patients was observed in an earlier Zimbabwean study where only 4 patients out of 58 (i.e., 7%) were shown to have signs of constriction over a longer observation period of 18 months [14]. In comparison a study done in South Africa in the 1980s presumably in HIV sero-negative individuals showed constriction in 11% of patients over a 2 year period [15]. Whilst the Zimbabwean study on HIV sero-positive patients did not however have sufficient power to substantiate this claim, we show that there may be external validity to the observation, provided that it is confirmed in appropriate studies.

We have found, in the first prospective observational study of the impact of HIV infection on the incidence of constrictive pericarditis following tuberculous pericardial effusion, that HIV infection is associated with a reduced incidence of the development of constrictive pericarditis. These observations suggest that the immune suppression associated with HIV reduces the risk of the development of pericardial fibrosis.
